# Modelling Predictors of Homophily on Perceived Oral Health Status Among Social Network Ties in a Population of Public Housing Residents

**DOI:** 10.1111/cdoe.13034

**Published:** 2025-02-26

**Authors:** Sharon M. Casey, Mabeline Velez, Robert McDonough, Julia C. Bond, Raul Garcia, Neha Gondal, Brenda Heaton

**Affiliations:** ^1^ Department of Health Policy & Health Services Research Boston University Henry M. Goldman School of Dental Medicine Boston Massachusetts USA; ^2^ Department of Epidemiology Boston University School of Public Health Boston Massachusetts USA; ^3^ Department of Sociology Boston University Boston Massachusetts USA; ^4^ Faculty of Computing & Data Sciences Boston University Boston Massachusetts USA; ^5^ University of Utah School of Dentistry Salt Lake City Utah USA

**Keywords:** Oral health, public housing, social network analysis

## Abstract

**Purpose:**

Individual behaviours are often shared within social networks (homophily), suggesting network‐level interventions hold promise for health promotion. Yet, little is known about oral health homophily. This study aimed to identify individual‐ and network‐based predictors of oral health homophily among individual's (ego) social networks of public housing residents.

**Methods:**

Respondents self‐reported demographics, oral health status and associated risk behaviours (*n* = 277). They named social contacts (alters), reported on relationship attributes, demographics and behavioural characteristics (*n* = 889). Hypothesised predictors of oral health homophily included relationship attributes (e.g., contact frequency), respondent‐level and shared characteristics. Oral health homophily was modelled using multilevel (hierarchical) logistic regression evaluating model attributes (AIC) to determine gains in explanatory power.

**Results:**

Relationship strength, including high frequency of shared meals and contact, was associated with higher odds of oral health homophily (OR [95% CI]: 1.92 [1.05, 3.52] and 1.62 [1.00, 2.63], respectively). The best performing model included daily shared meals and contact, respondent age, smoking and oral health status.

**Conclusions:**

Oral health homophily is predicted by relationship strength and ‘excellent/very good/good’ oral health. Respondents with poorer oral health and a smoking history were less homophilous in oral health. Multilevel interventions targeting oral health outcomes may benefit from accounting for social relationships.

**Abbreviations**
AIC: Akaike information criterion
CI: confidence interval
OR: odds ratio
US: United States

## 1 Introduction

More than 1 million households live in public housing communities across the United States (U.S) [1, 2]. Residents of public housing are predominantly of low socioeconomic position, members of racial/ethnic minority groups, and bear a disproportionately higher burden of disease, including oral diseases and poor oral health outcomes [3, 4]. Chronic oral health conditions have been associated with worse quality of life and serious long‐term consequences, including tooth loss, poor nutrition, frailty and social isolation [5, 6]. Notably, the burden of poor oral health is disproportionately experienced by those individuals and groups who are socially or economically disadvantaged.

Population disparities and inequities in oral health are the product of complex sociocultural factors, and their mitigation requires the use of complex systems science approaches [7]. Yet, interventions aimed at mitigating disparities in oral health have focused primarily on either individuals or entire communities, with little success [8, 9]. Given the geographic and social isolation of communities at the highest risk of poor oral health, there is growing evidence supporting intervention within the context of social networks, collections of relationships (such as family, friendship and formal) between human beings [10, 11]. The success of such interventions depends on the structural properties and composition of networks including ‘homophily’, the tendency for individuals to seek to interact or form relationships with others who are similar to them in terms of attributes and behaviours [12]. Homophily engenders clustering or pooling of behaviours among groups of interconnected persons in social networks including along adverse health outcomes such as alcohol consumption, smoking and suicidal ideation [13, 14, 15]. This homogeneity means less healthy individuals have fewer relatively healthy persons in their social networks, which impedes exposure to healthful behaviours and outcomes. Accordingly, targeting individual persons in interventions may be less effective than targeting collections of interconnected persons. Research supports that homophily facilitates the spread of information and/or resources in networks and is implicated in the reproduction of inequalities [16]. Furthermore, this type of transfer of knowledge and information is more likely among close relationships (such as those with family and close friends) due to trust and shared experience characterised by those ties [17].

Despite this evidence, few studies focus on homophily in a health context. Furthermore, although a prior study reports the presence of homophily on oral health status, little is known about determinants of health‐based homophily, a key piece of information needed for the design of network‐based health interventions [12]. Therefore, the primary aim of this egocentric social network study was to evaluate predictors of oral health homophily among a population of adults residing in public housing developments in Boston, MA. The objective was to identify individual (e.g., gender, education, behaviour) and relational (e.g., closeness, duration of relationship) determinants of homophily on oral health status by using personal network data as reported by the respondent (i.e., ‘ego’). Multilevel logistic regression was applied to develop a parsimonious predictive model of homophily on oral health in this egocentric network study.

## 2 Methods

### 2.1 Study Population and Participant Recruitment

Egocentric social network data were collected from residents of two government‐funded, public housing developments designated for families in Boston, MA. Adults (≥ 18 years) who spoke English and/or Spanish as their primary language, and who did not have plans to move from the housing development within the next 3 years, were considered eligible for participation. Recruitment occurred between March 2019 and March 2020 primarily through door‐to‐door knocking methods and the provision of study brochures for interested individuals to contact the research team. Prior work from this team has demonstrated the effectiveness of these recruitment methods for enrolling residents of public housing into research [18]. Verbal informed consent was obtained per the process approved by the Boston University Medical Campus Institutional Review Board (H‐38286).

### 2.2 Data Collection

Structured interviews were conducted in person either at the participant's home or in a community space. Interviewers (*n* = 3) were bilingual in English and Spanish and were all long‐standing members of the study team, well‐trained in interviewing techniques, including social network surveys. Responses were entered directly into a REDCap data collection form. Interviewers were observed and evaluated throughout the data collection period, with data entry quality checks built into the REDCap platform [19]. Interviews consisted of four modules: (1) respondent demographic characteristics and health history, (2) network generation in which participants were asked to list the names of people (i.e., ‘alters’) with whom they had discussed important matters, shared meals and regularly interacted within the public housing communities, (3) respondent reports of relationship attributes and individual characteristics and traits for each individual named and (4) respondent perceived reasons for relationships listed under each network generator, that is, important matters, shared meals and community contact.

### 2.3 Measures

The primary outcome of interest in this study is homophily on perceived oral health status. Egos (respondent) responded to a clinically validated self‐report measure of oral health status, and indicated their perception of each alter's (named individual) oral health status by responding to the question, ‘*Overall, how would you rate the health of your/[named alter's] teeth and gums…?'*’ using a Likert response scale of ‘Excellent’, ‘Very Good’, ‘Good’, ‘Fair’ or ‘Poor’ [20]. For analysis, response categories were collapsed to compare ‘Excellent/Very Good/Good’ to ‘Fair/Poor’. To determine the presence of homophily on perceived oral health status using this binary measure, the ego's oral health self‐rating was compared to their perception of alter oral health status; if the ego's report (based on the binary measure) matched their perception of the alter's oral health status (also based on the binary measure), the social network tie (ego‐alter connection) was considered homophilous on oral health status. If they did not match, the tie was deemed non‐homophilous on oral health status. Similarly, self‐ and ego‐reported general health status were assessed with the question ‘*Would you say your/[named alter's] health in general is…?*’ using the same original Likert response scale and collapsed according to the above.

#### 2.3.1 Individual Determinants

Self‐ and ego‐reported sociodemographic information of egos and alters was assessed, including age (years), gender (Female, Male, other gender identity), race/ethnicity (White/non‐Hispanic, Black/non‐Hispanic, Hispanic, non‐Hispanic), employment status (Full‐time, Part‐time, Occasional, Unemployed, Student, Homemaker, Retired), education level (No formal education, Less than high school, GED/High school diploma, Some/completed vocational/technical school, Some/completed college, Graduate/advanced degree) and current caregiver of young children (yes, no).

Oral health risk behaviours were assessed using self‐ and ego‐reported measures commonly used in public health surveillance. Egos self‐reported their sugar‐sweetened beverage and sugar‐sweetened food consumption patterns by responding to the following questions: ‘*How often do you drink sweet or sugary drinks* (*for example: juice, soda, pop, lemonade, Coke, Pepsi, Mountain Dew, Kool‐Aid, Gatorade*)?’ and ‘*How often do you eat sweet or sugary foods* (*for example: candy, cookies, donuts, ice‐cream*)?’. Egos also reported their perceptions of sugary food and beverage consumption patterns of alters. For self‐ and ego‐reported measures, responses were categorised as ‘Rarely or never,’ ‘Once a week’, and ‘Daily’, and further collapsed to ‘Daily’ and ‘Less than Daily’ for analysis. Cigarette smoking behaviours were categorised as ‘Daily’, ‘Non‐daily’, ‘Former smoker’, and ‘Never smoker’. Daily smoking was defined as smoking at least once daily, while a nondaily smoker was defined as someone who smokes from time to time. Response options were categorised as ‘Ever (current/former)’ and ‘Never’ for analysis. In all cases, if the ego's self‐reported response matched their report of the alter's perceived behaviour, the network tie (ego–alter connection) was considered homophilous; otherwise, the tie was deemed nonhomophilous.

#### 2.3.2 Relational Determinants

The strength of an ego–alter relationship (tie strength) was investigated by asking about specific attributes of the ego–alter relationship, as reported by the respondent. Frequency of shared meals was assessed by asking, ‘*In the past year, how often did you share meals or purchase food with or for [alter]?*’ and frequency of contact was assessed by asking: ‘*In the past year, how often did you see or talk to [alter]?*’. In both cases, responses included ‘Daily’, ‘Weekly’, ‘Less than every week’, ‘Rarely or never’, which were dichotomised to ‘Daily’ versus ‘Less than daily’ for analysis. Closeness of the relationship was assessed by asking: ‘*On a scale of 1 to 5, how close would you consider yourself to be to [alter's name]?*’ with responses ranging from ‘Not at all close’ to ‘Very close’ and dichotomised to ‘Very close’ compared to all other responses for analysis. The number of years known was reported and dichotomised (≤ 14 years and ≥ 14 years) for analysis. Fourteen years was chosen as the cut point for the duration of the relationship (‘years known’) as this was the average length of time participants reported having lived in their current public housing development. If we were to use the average duration of the relationship as the cut point, this would likely lead us to distinguish between family vs. other types of relationships or ties. Relationship type was categorised as ‘Friend or other’ (response options included acquaintance, coworker or associate, health professional, educator/teacher, religious leader/mentor) and ‘Family’ (response options included spouse/romantic partner, parent, sibling, child, other relative) for analysis.

### 2.4 Statistical Analysis

Observations with no reported alters were dropped from the analysis (*n* = 5), leaving a total analytic sample of 272. Descriptive analyses of ego and alter demographic information and characteristics were tabulated. Bivariate associations (Odds Ratio (OR), 95% confidence interval (CI)) between respondent‐reported measures and perceived oral health status homophily (yes vs. no) were computed. These measures included tie strength variables (Frequency of shared meals, Frequency of contact, Closeness of the relationship, Years known and Relationship type), ego‐level variables (Age, Education, Employment, Caregiver status, and Hispanic ethnicity), ego–alter homophily variables (Gender, Age difference, Hispanic ethnicity, Housing category, Caregiver status and Education category), as well as ego oral health risk behaviours (sugar‐sweetened beverage and food consumption, smoking status), ego health outcome variables (oral and general health status) and ego–alter homophily on sugar‐sweetened beverage and food consumption and smoking status.

The relationship between independent variables (tie strength, ego–alter homophily on demographics and oral health risk behaviours, ego oral health status and risk behaviours) and oral health homophily was modelled using multilevel (hierarchical) binary logistic regression with penalised quasi‐likelihood estimation. Alters were treated as nested within egos in these models. As ego‐reported perceptions of alter behaviours are likely to be correlated, it is not appropriate to treat each alter as independent of other alters mentioned by the same ego [21].

Variables occur at two levels: (1) ego (individual) and (2) ego–alter tie (relational). Therefore, several variables were used to measure relational effects on the outcome, including frequency of contact between ego and named alter, closeness of ties, frequency of shared meals, years known and relationship type (distinguishing between friends and others compared to family members). The associations between sociodemographic and behavioural homophily and perceived oral health homophily were also evaluated. At the ego level, sociodemographic variables including age, education level, employment status and race/ethnicity were included, as well as ego behavioural variables such as consumption of sugar‐sweetened beverages and food, smoking status and subjective oral and general health status evaluation.

To predict ego–alter oral health homophily, predictive model building was implemented using a theory‐based approach. Specifically, the strength of network ties [17] was hypothesised to be a significant relational determinant of oral health homophily, followed by ego‐level demographic variables and homophily on other oral health risk behaviours [12]. Therefore, forward selection methods were applied by first including those variables in each of the abovementioned categories based on an evaluation of the bivariate associations with the primary outcome, followed by an evaluation of the model attributes (model Akaike information criterion [AIC] values) to determine gains in explanatory power.

## 3 Results

Characteristics of the study population (*n* = 272) are presented in Table 1. Egos were predominantly female (84.6%), identified as Hispanic ethnicity (61.8%), and had a mean age of 51 years. Only 18% reported full‐time employment, and 35% reported less than a high school education. Among egos, 71.3% reported daily consumption of sugar‐sweetened beverages, while 29.4% reported daily consumption of sugary foods. The majority reported their smoking status as ‘never smoked’ (57.4%). Approximately half of egos reported their general health (55.2%) and oral health (50.8%) as ‘Excellent/very good/good’.

**TABLE 1:** Baseline ego‐reported sociodemographic characteristics and risk behaviours of egos and alters.

	Ego, *N* = 272	Alter, *N* = 889
	%	%
Age, years (mean, SD [range])	51, 14.4 (19–90)	48.6, 16 (18–97)
Gender—female^a^	84.6	74.5
Race/ethnicity		
White/non‐Hispanic	1.8	3.1
Black/non‐Hispanic	32.4	31.6
Hispanic	61.8	53.8
Non‐Hispanic	2.9	2.7
Employment		
Full‐time employment	18.4	—
Part‐time employment	23.9	—
Occasional employment	4.4	—
Unemployed	24.6	—
Unemployed; student	1.1	—
Unemployed; homemaker	18.0	—
Retired	9.6	—
Education level		
No formal education	0.7	1.2
Less than high school	34.9	17.3
GED/High school diploma	32.0	37.7
Some/completed vocational/technical school	4.8	8.7
Some/completed college	24.6	16.9
Graduate/advanced degree	2.6	2.5
Primary caregiver of a child under 6 years	18	16.4
Residence		
Same household	/	11.7
Same housing development	/	26.8
Another Boston housing development	/	6.3
Private residence in Boston	/	26.4
Elsewhere	/	27.3
Oral health status		
Excellent	9.9	15.4
Very good	8.5	14.1
Good	32.4	42.4
Fair	30.9	15.6
Poor	15.1	5.3
General health status		
Excellent	9.6	11.1
Very good	12.9	11.2
Good	32.7	44.9
Fair	35.7	24.1
Poor	8.8	6.4
Sugar‐sweetened beverage consumption		
Rarely or never	16.2	33.9
Once a week	12.5	8.2
Daily	71.3	49.8
Sugar‐sweetened food consumption		
Rarely or never	41.5	37.6
Once a week	29.0	12.9
Daily	29.4	42.0
Smoking status		
Current smoker	19.1	16.0
Former smoker	22.8	11.7
Never smoked	57.4	67.8

^a^ Ego: female vs. male *n* = 39, other/don't know/missing *n* = 3; Alter: female vs. male *n* = 221, other/don't know/missing *n* = 6.

The 272 (egos) in this study named 889 alters. The number of alters named by each ego ranged from 1 to 12, with an average of 3.3. 44% of alters were considered family. The average network density, calculated as the percentage of alter–alter ties divided by the number of possible ties within the ego network, is 0.51. On average, distributions of alter characteristics were similar to ego distributions with a few minor exceptions. On average, alters had more education (only 18.5% with less than high school education), consumed sugar‐sweetened beverages less frequently (50% vs. 71%), had never smoked (68% vs. 57%) and had better general and oral health (67.2% and 71.9% with ‘Excellent/Very good/Good’, respectively). Employment status was not assessed for alters. The proportion of ‘Don't know’/missing responses was generally infrequent aside from ego's perception of named alters' education status (15.7%), race/ethnicity (8.8%) and oral health status (7.2%).

Table 2 presents bivariate associations (OR, 95% CI) between oral health homophily (yes vs. no) and various individual and relational, ego‐reported measures. Among relational variables, indicators of tie strength, such as frequency of shared meals (daily vs. less than daily) was associated with higher odds of oral health homophily (OR 1.92, 95% CI 1.05, 3.52), followed by frequency of contact (daily vs. less than daily) (OR 1.62, 95% CI 1.0, 2.63). Among individual, ego‐level variables, older age was associated with decreased oral health homophily (OR 0.56, 95% CI 0.38, 0.82). Homophilous ties were less likely among egos who ever smoked (OR 0.31, 95% CI 0.14, 0.69). Those who reported better oral health were more likely to report ties who were homophilous on oral health (OR 31.03 [95% CI 14.78, 65.14]).

**TABLE 2:** Bivariate associations with oral health homophily (*n* = 889).

	Oral health homophily (yes vs. no)		
	OR (95% CI)	*N*	AIC values
Tie strength variables (relational)			
Frequency of shared meals (daily vs.< daily)	1.92 (1.05, 3.52)*	849	976.2
Frequency of contact (daily vs.< daily)	1.62 (1, 2.63)*	848	976.9
Closeness (very close vs. < very close)	1.59 (0.9, 2.83)	849	981.3
Years known (> 14 years vs. ≤ 14 years)	1.34 (0.85, 2.09)	851	985.2
Relationship type (family vs. friends/other)	1.36 (0.9, 2.07)	850	984.3
Ego‐level variables (individual)			
Age, years	0.56 (0.38, 0.82)**	849	972
Education (> high school vs. ≤ high school)	0.83 (0.38, 1.8)	846	978.7
Employment status (employed vs. unemployed)	2.25 (1.04, 4.9)*	852	982.5
Primary caregiver of a child < 6 years (yes vs. no)	2.69 (0.96, 7.59)	852	983.2
Hispanic ethnicity (yes vs. no)	1.46 (0.69, 3.1)	852	986
Ego–alter demographic homophily (relational)			
Gender (homophily vs. nonhomophily)	1.19 (0.75, 1.9)	846	977.4
Age difference (homophily vs. nonhomophily)	0.94 (0.73, 1.2)	835	963
Hispanic ethnicity (homophily vs. nonhomophily)	1.97 (0.88, 4.41)	852	984.5
Housing category (homophily vs. nonhomophily)	1.04 (0.69, 1.57)	852	986.9
Caregiver status (homophily vs. nonhomophily)	1.03 (0.66, 1.59)	845	980.9
Education level (homophily vs. nonhomophily)	1.4 (0.91, 2.16)	846	976.8
Ego‐level oral health risk behaviour and status (individual)			
Sugar‐sweetened beverages (daily vs. < daily)	1.3 (0.58, 2.92)	852	986.5
Sugar‐sweetened food (daily vs. < daily)	0.7 (0.32, 1.6)	852	986.3
Smoking status (ever vs. never)	0.3 (0.14, 0.69)**	844	969.1
Oral health status (excellent/very good/good vs. fair/poor)	31.0 (14.78, 65.14)***	849	861.4
Ego–alter oral health risk behaviour homophily (relational)			
Sugar‐sweetened beverages (homophily vs. nonhomophily)	1.46 (0.93, 2.3)	852	984.6
Sugar‐sweetened food (homophily vs. nonhomophily)	1.12 (0.7, 1.78)	851	986.2
Smoking status (homophily vs. nonhomophily)	1.03 (0.66, 1.61)	852	986.9

*Note:* Modelled as Index vs. Reference level for all variables. Significance levels: *p < 0.05, **p < 0.01, ***p < 0.001.

Abbreviations: AIC, Akaike information criterion; CI, confidence interval; OR, odds ratio.

Table 3 displays results from several progressively inclusive multivariable models that first include relational variables, followed by individual variables. Compared with the bivariate model statistics, a model inclusive of daily shared meals and social contact had improved fit (AIC = 970). Ego–alter ties in which there is a higher frequency of daily shared meals or social contact tend to be more homophilous on oral health (OR 1.87, 95% CI 0.98, 3.59 and OR 1.32, 95% CI 0.79, 2.21, respectively). Model fit continued to improve with the inclusion of ego age (years) and smoking status (ever vs. never), in which case homophilous ties on oral health were less likely as ego age increased and among egos who ever smoked. The final addition of ego oral health status resulted in the most parsimonious model (AIC = 827.6) and was indicative of a substantially higher odds of oral health homophily in cases where the ego reported being in 'Excellent/Very good/Good' oral health (OR 32.54, 95% CI 15.1, 70.2). The final model included both relational variables (e.g., frequency of shared meals and social contact), along with individual variables (e.g., ego age, smoking status and oral health status).

**TABLE 3:** Multivariable hierarchical models predicting oral health homophily (*n* = 889).

	Models, OR (95% CI)			
	*N* = 845	*N* = 843	*N* = 834	*N* = 831
**Relational variables** Frequency of shared meals				
Daily	1.87 (0.98, 3.59)	1.87 (0.97, 3.6)	1.85 (0.96, 3.58)	1.9 (1.02, 3.52)*
Less than daily	ref	ref	ref	ref
Frequency of contact				
Daily	1.32 (0.79, 2.21)	1.3 (0.78, 2.18)	1.31 (0.78, 2.21)	1.38 (0.84, 2.26)
Less than daily	ref	ref	ref	ref

Individual variables				
Age (years)		0.56 (0.37, 0.83)**	0.61 (0.41, 0.91)*	0.99 (0.73, 1.35)
Smoking status				
Ever (current/former)			0.36 (0.16, 0.82)*	0.49 (0.27, 0.91)*
Never			ref	ref
Oral health status				
Excellent/very good/good				32.54 (15.08, 70.19)***
Fair/poor				ref

Model AIC values	970	957.9	945	827.6

*Note:* Significance levels: *p < 0.05, **p < 0.01, ***p < 0.001.

Abbreviations: AIC, Akaike information criterion; CI, confidence interval; OR, odds ratio.

## 4 Discussion

Relational variables were consistently associated with perceived oral health homophily. The strongest associations were observed for the frequency of shared meals and contact, with a higher frequency of both associated with a higher prevalence of ego–alter ties that were reported to be homophilous on oral health. These findings suggest that close relationships, defined by frequent interaction, are more likely to be homophilous on oral health status. Additionally, egos who had ever smoked were more likely to perceive alters as having nonhomophilous oral health status, whereas egos with excellent/very good/good oral health themselves were more likely to perceive their alters as having comparable oral health.

These results have implications for multilevel interventions that primarily leverage the social network [22]. Meal sharing, for example, generated ego–alter ties that had a higher frequency of reported homophily on oral health status. Given the wealth of evidence pointing to the dietary causes of poor oral health, [23] interventions that centre on meal sharing may present an opportunity to improve upon the impact of interventions that aim to target the dietary causes of poor oral health on an individual level only. Findings also indicate that better oral health tends to cluster within social networks in which the ego reported having excellent‐to‐good oral health. This may imply that those individuals reporting better oral health may benefit from an added layer of social advantage beyond those in poor oral health [24]. If this is the case, the observed phenomena may be an important contributor to the occurrence of oral health inequities, warranting the use of multilevel perspectives. Furthermore, the frequency of oral health homophily among egos who report excellent‐to‐good oral health was more than 30 times higher than that of egos who reported fair‐to‐poor oral health, indicating that individuals with unfavourable oral health may be forming nonhomophilous relationships to access needed resources and/or information [11, 12, 25, 26]. Although this could only be verified with longitudinal social network evaluations, one could reasonably assume that the impact of oral health promotion interventions may be strengthened by targeting close ties who were nonhomophilous on oral health, given these findings.

This study evaluates data from public housing developments managed by the Boston Housing Authority. These housing communities often share features with other developments across the United States; thus, findings from this study may be generalisable to similar urban housing communities across the United States. While findings from this research provide valuable insights into individual‐level and network‐based attributes predictive of homophily on oral health status, there are several limitations to consider. First, alter‐level data are reported as perceived by the egos, which may result in bias. If egos project their personal oral health status and/or risk behaviours onto that of their named alters, there is a general expectation of upward bias in estimates of the outcome under study, oral health status homophily [12]. However, investigations report that an ego's perception of alter characteristics is often quite accurate, especially regarding observable traits and health characteristics [27, 28]. A second limitation of this study is that data are gathered from just two public housing developments within Boston. Though these housing developments share characteristics with other, similar developments in Boston, MA, and the United States more broadly, there may be distinct features of these developments that may influence these findings. It is also important to consider that data in this study are limited to the types of ‘name generator’ questions applied to elicit names of alters. A more expansive list of ‘name generator’ questions may result in identifying a larger social network. However, the questions asked in this study to identify social networks are based on a theoretical framework to identify tie strength; for example, the frequency of shared meals between an ego and an alter.

Oral health disparities and inequities continue to persist throughout the United States. The study of social networks and the attributes of social ties in relation to oral health holds promise for developing multilevel approaches to addressing population oral health. Findings from this study identify key predictors of oral health homophily that lend novel insights into potential mechanisms that underlie the observed disparities and offer opportunities for innovative intervention approaches.

## Conflicts of Interest

The authors declare no conflicts of interest.

## Data Availability

The data that support the findings of this study are available from the corresponding author upon reasonable request.
